# UBA1 as a proteostasis-innate immunity checkpoint: lessons from vexas syndrome and therapeutic implications

**DOI:** 10.3389/fimmu.2026.1974344

**Published:** 2026-09-22

**Authors:** Yanyu Zhang, Quanxin Su

**Affiliations:** First Affiliated Hospital, Dalian Medical University, Dalian, China

**Keywords:** clonal hematopoiesis, innate immunity, proteostasis, TAK-243, uba1, ubiquitination, unfolded protein response, VEXAS syndrome

## Abstract

Ubiquitin-like modifier activating enzyme 1 (UBA1) occupies the apex of the canonical ubiquitination cascade and supplies activated ubiquitin to a broad network of E2 conjugating enzymes and E3 ligases. Although traditionally regarded as an essential housekeeping enzyme, human genetics has revealed a more instructive function for UBA1: it determines whether proteostatic stress is buffered, converted into inflammatory adaptation, or progresses to cell death. Somatic UBA1 mutations causing VEXAS syndrome provide a natural experiment in this threshold biology. Partial and compartment-selective loss of UBA1 activity permits hematopoietic cells to survive while reducing ubiquitination capacity, activating stress-response pathways, altering cell-death thresholds, and promoting innate immune dysregulation. Single-cell and functional studies further demonstrate early inflammatory activation in UBA1-mutant hematopoietic progenitors, myeloid-biased differentiation, monocyte dysfunction, and inflammasome-associated signaling. Conversely, profound pharmacological UBA1 inhibition with agents such as TAK-243 drives proteotoxic collapse and is being explored as an anticancer strategy. Here, we propose that UBA1 functions as a proteostasis-innate immunity checkpoint whose biological output is determined by residual enzymatic activity, subcellular distribution, lineage context, and clone size. This framework reconciles inflammatory disease caused by partial UBA1 dysfunction with the antitumor effects of UBA1 inhibition and suggests a mechanism-based approach to therapeutic intervention in VEXAS syndrome and malignancy.

## 1 Introduction

Ubiquitination is commonly introduced as a mechanism of protein degradation, but its biological reach is substantially broader. Ubiquitin-dependent signaling regulates protein quality control, receptor trafficking, DNA repair, cell-cycle progression, organelle homeostasis, inflammatory signaling, and cell death. At the apex of this system is ubiquitin-like modifier activating enzyme 1 (UBA1), the predominant ubiquitin E1 enzyme in mammalian cells. Although UBA6 can also activate ubiquitin, it has a more restricted functional repertoire and does not substitute for the broad ubiquitin-activating role of UBA1. UBA1 activates ubiquitin through ATP-dependent adenylation, forms a high-energy UBA1-ubiquitin thioester intermediate, and subsequently transfers ubiquitin to multiple E2 enzymes that feed diverse downstream ubiquitin circuits (1–6).

The essential nature of UBA1 long suggested a simple biological model: severe loss of activity should be lethal, whereas modest reductions would be tolerated. The discovery of VEXAS syndrome challenged this view. VEXAS (vacuoles, E1 enzyme, X-linked, autoinflammatory, somatic) is a late-onset inflammatory and hematologic syndrome caused by acquired somatic mutations in the X-linked UBA1 gene in hematopoietic stem and progenitor cells (HSPCs). Clinically, it is characterized by systemic inflammation with manifestations such as chondritis, neutrophilic dermatoses, pulmonary involvement, and thrombosis, together with macrocytic anemia, other cytopenias, and bone marrow abnormalities (7–10). This combination of inflammatory, hematologic, and somatic genetic features makes VEXAS relevant across rheumatology, hematology, oncology, and genetics. Importantly, VEXAS is not caused by complete absence of UBA1. Instead, disease-associated mutations frequently create a state of partial and compartment-selective UBA1 dysfunction in which mutant hematopoietic cells remain viable but operate with reduced ubiquitination capacity. UBA1 dysfunction is also associated with clinically distinct disorders: germline variants cause X-linked spinal muscular atrophy type 2 (SMAX2), whereas somatic UBA1 variants have also been identified in lung cancer in never-smokers. Comparative mechanistic studies show that VEXAS-associated p.Met41 mutations primarily alter cytoplasmic UBA1 isoform expression, whereas other disease-associated variants impair UBA1 through distinct effects on catalytic function or protein stability, underscoring how the affected isoform or functional domain and the resulting biochemical defect can shape disease phenotype (11). This observation raises a broader immunological question: how does impaired protein homeostasis become translated into sustained innate immune activation? Recent studies indicate that the answer cannot be reduced to a single cytokine or inflammasome. UBA1-mutant cells show abnormalities in proteostatic stress, unfolded protein response (UPR) signaling, inflammatory transcription, lineage selection, monocyte function, and cell-death regulation (12–15). These processes occur at different levels of hematopoiesis and may be mechanistically coupled without being strictly linear.

Building on this clinical and genetic context, we use VEXAS syndrome as a human perturbation model revealing a previously underappreciated function of UBA1. We propose that UBA1 acts as a proteostasis-innate immunity checkpoint. Within this framework, preserved UBA1 activity maintains homeostasis, partial dysfunction generates a viable stress-adapted and inflammation-prone state, and profound inhibition causes proteostatic collapse and cell death. This threshold model provides a mechanistic bridge between autoinflammation, clonal hematopoiesis, and pharmacological UBA1 targeting.

## 2 UBA1 defines a cellular proteostatic threshold

### 2.1 An upstream bottleneck in ubiquitin signaling

UBA1 is unusual among components of the ubiquitin system because of its upstream position. Whereas individual E3 ligases act on restricted groups of substrates, perturbing UBA1 potentially changes ubiquitin loading across numerous E2-E3 networks simultaneously. Structural and biochemical studies have shown that ubiquitin activation requires coordinated rearrangement of the adenylation, catalytic cysteine, and ubiquitin-fold domains and tightly controlled transfer to E2 enzymes, allowing UBA1 to perform chemically distinct activation and transthiolation reactions within a single catalytic cycle (2–6).

Human UBA1 is also compartmentalized. Early biochemical work established nuclear and cytoplasmic pools of E1 and demonstrated regulated nuclear targeting (16–18). The longer UBA1a isoform is enriched in the nucleus, whereas translation initiated at Met41 generates the predominantly cytoplasmic UBA1b isoform. Canonical VEXAS-associated Met41 substitutions impair normal UBA1b production and favor expression of a catalytically compromised cytoplasmic species while substantially preserving nuclear UBA1 activity (8). This provides a mechanistic explanation for an otherwise paradoxical phenotype: mutations in an essential enzyme can produce severe disease without causing universal cell death.

The expanding spectrum of non-Met41 mutations further indicates that ‘UBA1 deficiency’ is not a single biochemical condition. Splice-site variants, S56F, recurrent active-adenylation-domain substitutions, and other non-Met41 variants can alter protein abundance or catalytic function through mechanisms distinct from the canonical isoform switch (9, 10, 19–22). Residual enzyme activity, cellular compartment, and downstream E2 engagement are therefore likely to determine phenotype at least as strongly as the presence of a UBA1 mutation itself. In particular, impaired ubiquitin transfer from UBA1 to E2 enzymes can create an E2-charging bottleneck that constrains downstream ubiquitin signaling (11).

### 2.2 Partial loss of ubiquitination generates adaptation

Reduced ubiquitination should not be considered synonymous with impaired proteasomal degradation. Ubiquitin controls processes extending from ribosome quality control and organelle homeostasis to DNA-damage signaling. Importantly, experimental reduction of UBA1 or E2 activity can trigger compensatory programs that preserve selected cellular functions despite decreased ubiquitination, whereas UBA1 depletion also disrupts coordinated DNA-damage responses (1, 23, 24). Reduced ubiquitin capacity can therefore elicit selective, function-dependent adaptive responses rather than uniform compensation across UBA1-dependent pathways.

This observation provides a useful conceptual framework for VEXAS. Near-complete inhibition of UBA1 rapidly depletes ubiquitin conjugates and becomes cytotoxic. Mild reductions may be compensated. Between these extremes lies an intermediate state in which residual UBA1 activity is sufficient for survival but insufficient to maintain an unstressed proteome.

We propose that this intermediate state is critical to inflammatory disease. Persistent imbalance between protein production, folding, trafficking, and disposal places cells under chronic proteotoxic pressure. Rather than immediately dying, susceptible hematopoietic cells activate survival programs, remodel metabolism, and modify inflammatory and cell-death thresholds. The biological consequence is therefore not simply ‘less ubiquitination,’ but a new cellular state shaped by chronic adaptation.

## 3 From proteostasis failure to innate immune rewiring

### 3.1 The unfolded protein response as an immune interface

The UPR provides one of the clearest mechanisms through which proteostatic stress can influence immunity. Its three canonical branches-PERK, IRE1, and ATF6-initially function to restore endoplasmic reticulum (ER) homeostasis by reducing protein synthesis, increasing folding capacity, and promoting removal of damaged proteins. Under persistent stress, however, the same pathways can reshape inflammatory transcription, metabolism, reactive oxygen species production, and cell-death susceptibility (25–28).

In immune cells, the UPR is extensively integrated with Toll-like receptor (TLR) signaling, NF-κB, interferon pathways, and cytokine production (26, 28). Consequently, chronic ER stress can become an active regulator of immune phenotype rather than simply a downstream marker of cellular injury.

Single-cell studies of VEXAS bone marrow support this connection. UBA1-mutant HSPCs display enrichment of stress-response and inflammatory pathways, with inflammatory activation detectable early in hematopoietic differentiation and particularly prominent in mutant myeloid populations (13, 14). These findings suggest that inflammatory programming is not solely imposed on mature cells by the extracellular cytokine environment. At least part of the phenotype originates cell intrinsically within the mutant hematopoietic hierarchy.

An important unresolved issue is whether UPR activation is primarily pathogenic or adaptive. It may be both. Stress pathways may enable mutant progenitors to survive reduced ubiquitination while simultaneously creating a transcriptional environment permissive for inflammation. In this model, the UPR is not merely a bridge from protein damage to inflammation; it may also provide the fitness required for propagation of the mutant clone.

### 3.2 Inflammasome activation is a component, not the entire mechanism

Monocytes appear to represent an important effector compartment in VEXAS. Functional and multi-omic analysis have demonstrated abnormal monocyte states together with increased circulating IL-1β and IL-18 and evidence of inflammasome activation (14). Proteotoxic stress provides several plausible routes to inflammasome sensitization, including mitochondrial dysfunction, oxidative stress, ion flux, and accumulation of damage-associated signals.

However, a purely inflammasome-centric model is insufficient. VEXAS transcriptomic studies also identify TNF, NF-κB, interferon, and other inflammatory programs (13, 14). Moreover, clinical responses to individual cytokine-blocking strategies are variable. These observations favor a network model in which inflammasome activation represents one downstream node of a broader proteostasis-driven inflammatory state.

The distinction has therapeutic relevance. Blocking IL-1 signaling may suppress one important output without correcting the underlying defect in ubiquitin activation, stress adaptation, or clonal hematopoiesis. This may help explain why downstream anti-inflammatory therapy frequently reduces symptoms without producing durable molecular remission.

### 3.3 Altered cell-death thresholds may amplify inflammation

UBA1 dysfunction also influences whether stressed cells survive or die. Experimental VEXAS models indicate altered sensitivity to inflammatory cell-death programs and to direct UBA1 inhibition (29, 30). Cell death may therefore serve as an amplifier rather than the final endpoint of UBA1 dysfunction: dying cells release danger-associated signals that activate neighboring innate immune cells, while inflammatory cytokines impose additional stress on mutant hematopoietic populations.

This creates a potential feed-forward circuit in which reduced ubiquitin capacity promotes proteotoxic stress, stress adaptation and inflammatory priming, inflammatory cell death, release of danger signals, and further innate immune activation.

The same upstream mutation may therefore have different consequences at different developmental stages. Primitive progenitors capable of mounting effective adaptive stress responses may persist and expand, whereas differentiated myeloid cells exposed to additional inflammatory signals may more readily cross a cell-death threshold. This lineage-dependent behavior offers one explanation for the coexistence of clonal persistence and tissue-damaging inflammation.

## 4 VEXAS reveals lineage- and clone-dependent checkpoint biology

The hematopoietic architecture of VEXAS provides strong evidence that cellular context modifies the effects of UBA1 dysfunction. UBA1-mutant alleles are commonly enriched in myeloid lineages, whereas mature lymphoid compartments show substantially lower representation. Single-cell and clonal studies demonstrate granulocytic and monocytic bias together with altered lymphoid representation (13, 15, 31). Thus, a UBA1 mutation does not confer equivalent fitness across hematopoietic lineages.

Clinical cohorts reinforce that this lineage biology is expressed as a multisystem syndrome rather than an isolated marrow defect. Hematologic abnormalities, systemic inflammation, skin and cartilage disease, pulmonary involvement, thrombosis, and other organ manifestations recur across independent cohorts, although their combinations vary substantially between patients (31–34). An unresolved question is how UBA1-mutant hematopoiesis achieves clonal dominance despite evidence of impaired proliferative capacity and increased apoptotic programs in VEXAS HSPCs. Wu et al. highlighted this apparent discordance between mutant-clone dominance *in vivo* and impaired hematopoietic growth ex vivo, suggesting that clonal fitness cannot be inferred from proliferative capacity alone (13).

Several mechanisms may contribute to this apparent paradox. Myeloid progenitors may be more capable of tolerating reduced ubiquitination, while lymphoid progenitors may undergo increased apoptosis. Stress-response programs could differentially support mutant myelopoiesis. Inflammation itself may further favor myeloid production, although current evidence suggests that inflammatory activation and lineage bias are not necessarily the same biological process.

This distinction changes the interpretation of VEXAS therapy. Suppression of cytokines may improve fever, chondritis, and inflammatory markers without substantially reducing the mutant clone. Conversely, clone-directed therapy may change hematopoietic composition before all inflammatory outputs normalize. Clinical response and molecular response should therefore be regarded as related but non-equivalent endpoints.

Clone size is emerging as an additional dimension. Population and biobank studies indicate that pathogenic UBA1 variants can be detected across a wider spectrum of penetrance and variant allele fraction (VAF) than initially recognized (35–37). A 2026 nationwide prospective study further found that higher peripheral-blood UBA1 VAF was independently associated with poor outcomes, including death or transfusion dependence (38). However, earlier clinical data did not identify a significant association between UBA1 VAF and survival (8), indicating that the prognostic value of VAF is not uniform across cohorts. VAF may therefore be most informative when interpreted alongside genotype, hematologic phenotype, and other clinical risk factors rather than as an isolated prognostic measure.

At the same time, genotype matters. Met41 substitutions and noncanonical variants can produce different biochemical consequences, and emerging cohorts suggest genotype-phenotype relationships in inflammatory burden, hematologic manifestations, and prognosis (19–22, 37, 38). The clinically relevant variable may therefore not be UBA1 mutation status alone but an interaction among residual UBA1 activity, lineage context, mutant clone size, and host stress exposure.

The marrow phenotype provides a second readout of this checkpoint failure. Macrocytic anemia, cytopenias, hypercellularity, vacuolated myeloid and erythroid precursors, and variable dysplasia are repeatedly observed, but vacuolization is not entirely specific and should be interpreted with molecular testing and clinical context (31, 32, 39–43). VEXAS therefore represents a somatically acquired autoinflammatory condition at the interface of clonal hematopoiesis, inflammatory selection, and myeloid disease, distinct from the conventional paradigm of inherited monogenic autoinflammatory disorders (15, 44).

## 5 A three-state model of the UBA1 checkpoint

Taken together, these observations support a three-state model of the UBA1 checkpoint (**Figure 1**; **Table 1**). In the homeostatic state, preserved UBA1 capacity sustains ubiquitin flux, proteome surveillance, and transient stress buffering. In an intermediate stress-adapted state, partially reduced or compartment-selective UBA1 function remains compatible with survival but shifts cells toward chronic proteotoxic stress, UPR engagement, inflammatory priming, lineage selection, and altered cell-death thresholds; VEXAS syndrome provides the clearest human example of this state (8, 13, 14, 23). Beyond a lower viability threshold, profound UBA1 inhibition causes a broad loss of ubiquitin conjugation, overwhelming proteotoxic and DNA-damage stress, and apoptosis (24, 56). These states should not be interpreted as fixed biochemical cutoffs. Their position is likely shaped by lineage, baseline proteotoxic and metabolic load, inflammatory exposure, clone size, and compensatory ubiquitin pathways. This continuum explains how perturbation of the same upstream enzyme can favor chronic inflammatory adaptation in one context and cytotoxic collapse in another.

**Figure 1:**
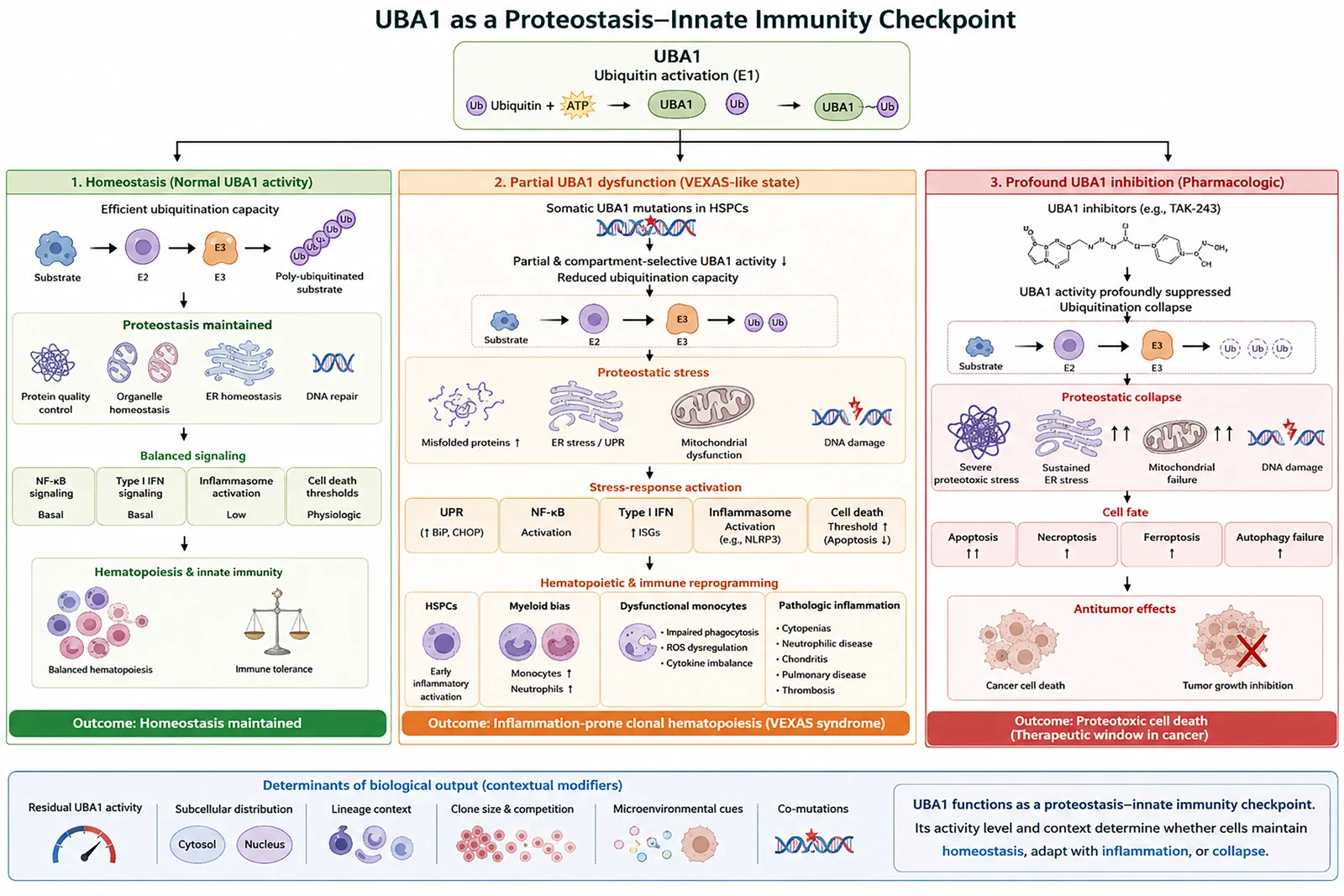
UBA1 as a proteostasis–innate immunity checkpoint. Preserved UBA1 activity maintains ubiquitin conjugation, proteome surveillance, and immune homeostasis. Partial or compartment-selective dysfunction, as in VEXAS syndrome, reduces ubiquitination capacity and promotes proteotoxic stress, UPR activation, inflammatory rewiring, inflammasome signaling, myeloid-biased hematopoiesis, and altered cell-death thresholds. Profound pharmacologic UBA1 inhibition crosses the viability threshold, causing proteostatic collapse and apoptosis, thereby creating a therapeutically exploitable vulnerability in stressed malignant cells. UBA1, ubiquitin-like modifier activating enzyme 1; VEXAS, vacuoles, E1 enzyme, X-linked, autoinflammatory, somatic; E1, ubiquitin-activating enzyme; Ub, ubiquitin; ATP, adenosine triphosphate; E2, ubiquitin-conjugating enzyme; E3, ubiquitin ligase; ER, endoplasmic reticulum; DNA, deoxyribonucleic acid; UPR, unfolded protein response; NF-κB, nuclear factor kappa B; IFN, interferon; HSPCs, hematopoietic stem and progenitor cells; BiP, binding immunoglobulin protein; CHOP, C/EBP homologous protein; ISGs, interferon-stimulated genes; NLRP3, NLR family pyrin domain containing 3; ROS, reactive oxygen species. **↑**, increased; **↓**, decreased.

**Table 1:** Three-state model of the UBA1 proteostasis-innate immunity checkpoint.

State	UBA1/ubiquitin capacity	Dominant cellular program	Immunological consequence	Biological/clinical analogy	Therapeutic implication
I. Homeostatic	Preserved	Efficient proteome surveillance; transient stress adaptation	Appropriate initiation and termination of immune signaling	Normal cells	No intervention
II. Stress-adapted	Partially reduced	Chronic proteotoxic stress; UPR engagement; lineage selection; altered death thresholds	Innate immune priming and cytokine amplification	Many VEXAS-associated UBA1 states	Suppress inflammatory output and/or selectively reduce the mutant clone
III. Proteostatic collapse	Severely reduced	Loss of ubiquitin conjugation; overwhelming ER and proteotoxic stress; DNA-damage response failure; apoptosis	Cell loss; secondary inflammation may occur	Potent pharmacological UBA1 inhibition	Exploit selectively in vulnerable malignant cells

The table summarizes the proposed continuum of UBA1 activity from homeostasis through partial dysfunction to profound inhibition, together with the corresponding cellular, immunological, and therapeutic consequences.

UBA1, ubiquitin-like modifier activating enzyme 1; UPR, unfolded protein response; ER, endoplasmic reticulum; DNA, deoxyribonucleic acid; VEXAS, vacuoles, E1 enzyme, X-linked, autoinflammatory, somatic.

## 6 Therapeutic leverage across the checkpoint

### 6.1 Controlling downstream inflammatory output

Glucocorticoids remain highly effective for rapid control of VEXAS inflammation but frequently lead to dependence and cumulative toxicity. JAK inhibitors and cytokine-directed biologics targeting IL-6, IL-1, and TNF have therefore been used as steroid-sparing approaches. Among these biologics, IL-6 blockade has shown the most consistent activity, whereas responses to IL-1 inhibition are more variable and TNF inhibition has generally shown limited efficacy in retrospective cohorts and meta-analysis (46–49). Importantly, cytokine suppression may not fully correct the underlying inflammatory state: Adachi et al. demonstrated persistent monocyte and neutrophil cell death and damage-associated signaling despite apparent clinical control with glucocorticoids and IL-6 receptor blockade, suggesting that cell death itself may continue to amplify inflammation (29). Infection and treatment-related toxicity remain important considerations in therapeutic selection (45, 48, 49).

From the checkpoint perspective, these therapies mainly attenuate output. They do not restore normal UBA1 activity and generally do not eradicate mutant hematopoiesis. This distinction may explain both their clinical utility and their limited disease-modifying capacity.

### 6.2 Targeting the mutant hematopoietic clone

Azacitidine provides a conceptually different strategy. Initial reports suggested inflammatory and hematologic benefit (50), and the large FRENVEX study subsequently demonstrated inflammatory, hematologic, and molecular responses, including reduction of UBA1-mutant burden in a substantial subset of patients (51). A 2026 systematic review of hypomethylating agents supports this clone-directed signal while highlighting toxicity, variable durability, and the need for prospective predictors of response (52). Molecular response is particularly important because it supports the principle that clinical improvement can result from contraction of the disease-driving clone rather than cytokine suppression alone.

Allogeneic hematopoietic stem cell transplantation goes further by replacing mutant hematopoiesis. A multicenter European Society for Blood and Marrow Transplantation study demonstrated the feasibility of durable clinical and molecular remission in selected patients, although transplant-related toxicity, graft-versus-host disease, infection, advanced age, and comorbidity remain major constraints (53).

These observations suggest a therapeutic hierarchy: downstream immunomodulation reduces inflammatory consequences, clone-directed therapy reduces the source of abnormal hematopoiesis, and transplantation can potentially replace it completely. The 2026 American College of Rheumatology guidance and the contemporary VEXAS disease primer both emphasize individualized, multidisciplinary management because treatment evidence remains dominated by retrospective studies rather than randomized trials (54, 55).

### 6.3 Deliberately collapsing the checkpoint in cancer

UBA1 biology has the opposite therapeutic implication in malignant cells. Cancer cells often carry high proteotoxic, replicative, and DNA-damage stress and may therefore operate closer than normal cells to the threshold beyond which reduced ubiquitination becomes lethal.

TAK-243 is a mechanism-based UBA1 inhibitor that markedly decreases ubiquitin conjugation and induces proteotoxic and DNA-damage stress. The first-in-class pharmacologic study established its mechanism and antitumor activity (56). Subsequent disease-focused studies demonstrated activity against acute myeloid leukemia and leukemia stem cells, B-cell lymphoma with ER-stress/UPR dependence, and small-cell lung cancer, including synergy with genotoxic therapies in preclinical models (57–59).

The conceptual symmetry is notable. In VEXAS, partial UBA1 dysfunction produces a viable but inflammatory state and treatment seeks either to suppress its outputs or remove adapted mutant cells. In cancer, pharmacological treatment attempts to push stressed malignant cells in the opposite direction-from compensated stress to irreversible proteostatic collapse.

Direct UBA1 inhibition in VEXAS itself is therefore intriguing but potentially hazardous. Recent primary-cell data show that VEXAS CD34+ cells can be more sensitive to TAK-243 than myelodysplastic or healthy CD34+ cells, providing proof of principle for a therapeutic window but not yet clinical validation (60). Killing large numbers of inflammatory myeloid cells could also intensify cytokine release or tissue damage, so any such strategy would require careful definition of dose, lineage specificity, and inflammatory consequences.

## 7 Discussion

The discovery of VEXAS has transformed UBA1 from a largely biochemical component of the ubiquitin cascade into a mechanistic link between proteostasis, clonal hematopoiesis, and innate immunity. The central lesson is that reduced ubiquitination is not a binary event. Its consequences depend on magnitude and context.

Malignancy represents another potentially important dimension of this context. Hematologic neoplasms are well recognized in VEXAS, and recent registry data also suggest an increased risk of both hematologic and non-hematologic malignancies. Several non-exclusive mechanisms may contribute: chronic inflammation and clonal selection could favor neoplastic evolution, while impaired UBA1-dependent DNA-damage responses may increase genomic vulnerability (21, 24, 31); however, a direct oncogenic role of VEXAS-associated UBA1 mutations remains unproven. Conversely, low-burden UBA1-mutant clones can be clinically silent, raising the possibility that malignancy-associated inflammatory or cellular stress could alter clone fitness or inflammatory output and contribute to disease emergence, although this reverse-causality model remains speculative. The relationship between VEXAS and malignancy may therefore be bidirectional but is currently mechanistically unresolved (37, 61, 62).

First, the relevant ubiquitinated substrates remain poorly defined. Global reduction in ubiquitination alone cannot explain lineage specificity or the diversity of inflammatory phenotypes. Quantitative ubiquitinomics in genotype-defined primary HSPCs and myeloid cells will be required to identify UBA1-sensitive E2/E3 modules and substrates that connect reduced ubiquitin activation to immune signaling.

Second, temporal causality remains uncertain. Inflammatory transcription, proteotoxic stress, myeloid bias, and clone expansion are all detectable in VEXAS, but their precise sequence is incompletely established. Longitudinal single-cell approaches integrating genotype, transcriptome, chromatin accessibility, proteomics, and ubiquitin modification should help distinguish initiating events from adaptive consequences.

Third, therapeutic trials should distinguish clinical, hematologic, and molecular response. Normalization of C-reactive protein or glucocorticoid reduction does not necessarily indicate contraction of the UBA1-mutant clone. Variant allele fraction, lineage-specific clone burden, inflammatory signatures, and proteostatic biomarkers should therefore be incorporated as parallel endpoints.

Fourth, current evidence suggests that the UBA1 checkpoint has a quantitative component. Biobank analysis demonstrate variable penetrance across VAF ranges, while the association between VAF and clinical outcomes has differed across cohorts (8, 35–38). VAF should therefore be interpreted longitudinally and in conjunction with genotype and clinical context rather than as a standalone prognostic marker.

Finally, the relevance of UBA1 extends beyond VEXAS. Mechanistic studies across UBA1-associated disorders and pharmacological inhibition show that the biological consequences of perturbing this enzyme depend on residual activity and cellular context (11, 23, 56–60). The important variable may therefore be neither ‘UBA1 deficiency’ nor ‘UBA1 inhibition’ in isolation, but the position of an individual cell relative to its proteostatic viability threshold.

We propose that UBA1 should be viewed as a proteostasis-innate immunity checkpoint: sufficient activity preserves homeostasis; partial dysfunction permits stress-adapted survival with inflammatory rewiring; and severe inhibition produces proteostatic collapse. VEXAS syndrome provides the clearest human evidence for the intermediate state. Defining the molecular thresholds that separate these states could enable therapies that suppress inflammation, selectively eliminate pathogenic clones, or exploit proteostatic vulnerability in cancer.
